# Lung Pathology of Mutually Exclusive Co-infection with SARS-CoV-2 and *Streptococcus pneumoniae*

**DOI:** 10.3201/eid2703.204024

**Published:** 2021-03

**Authors:** Tetsuya Tsukamoto, Noriko Nakajima, Aki Sakurai, Masayuki Nakajima, Eiko Sakurai, Yuko Sato, Kenta Takahashi, Takayuki Kanno, Michiko Kataoka, Harutaka Katano, Mitsunaga Iwata, Yohei Doi, Tadaki Suzuki

**Affiliations:** Fujita Health University School of Medicine, Toyoake, Japan (T. Tsukamoto, A. Sakurai, M. Nakajima, E. Sakurai, M. Iwata, Y. Doi);; National Institute of Infectious Diseases, Tokyo, Japan (N. Nakajima, Y. Sato, K. Takahashi, T. Kanno, M. Kataoka, H. Katano, T. Suzuki)

**Keywords:** coronavirus disease, COVID-19, autopsy, severe acute respiratory syndrome coronavirus 2, SARS-CoV-2, *Streptococcus pneumoniae*, co-infection, bronchopneumonia, acute respiratory distress syndrome, ARDS, viruses, bacteria, streptococci

## Abstract

Postmortem lung pathology of a patient in Japan with severe acute respiratory syndrome coronavirus 2 infection showed diffuse alveolar damage as well as bronchopneumonia caused by *Streptococcus pneumoniae* infection. The distribution of each pathogen and the accompanying histopathology suggested the infections progressed in a mutually exclusive manner within the lung, resulting in fatal respiratory failure.

Coronavirus disease (COVID-19), caused by severe acute respiratory syndrome coronavirus 2 (SARS-CoV-2) (*1*), has claimed >1 million lives worldwide (*2*). Respiratory failure is the leading cause of death from COVID-19; however, the pathogenic process of the combined infection of SARS-CoV-2 and other respiratory pathogens is not fully understood.

We describe the clinical course and postmortem pathologic findings of a patient in Japna who died from SARS-CoV-2 and *Streptococcus pneumoniae* co-infection. Extensive histopathologic and molecular analyses of the lungs and other organs provided insights into the pathogenesis of severe lung disease caused by the co-infection.

## Case Report

In March 2020, an 84-year-old man was brought to the emergency department at Fujita Health University Hospital (Toyoake, Japan) in cardiopulmonary arrest; his death was confirmed 20 minutes after he arrived at the hospital. He was found to have been in close contact with persons with confirmed SARS-CoV-2 cases at the adult day care center he attended and had been in self-isolation at home for 5 days before his death. He had been in generally good health until 8 days before his arrival at the hospital, when he developed sore throat and fatigue. Four days later, he developed a cough and lost his appetite. A whole-body computed tomography scan performed at the hospital showed bilateral diffuse consolidation with ground-glass opacities in the lungs and no gross abnormality in the other organs (Appendix Figure 1). SARS-CoV-2 infection was diagnosed after his death by real-time reverse transcription PCR (rRT-PCR) of a nasopharyngeal swab specimen. The family gave consent for an autopsy to be performed.

**Figure 1:**
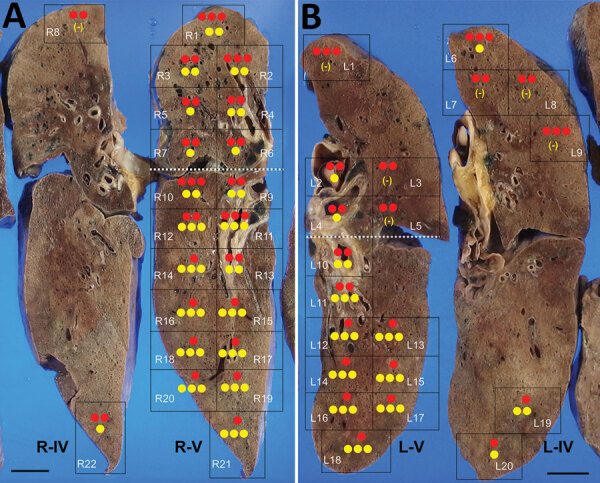
Molecular detection of SARS-CoV-2 and *Streptococcus pneumoniae* in the lungs of a patient in Japan co-infected with both pathogens. The 42 lung sections were analyzed and the amount of SARS-CoV-2 RNA and *S. pneumoniae* DNA in each section was evaluated. A) The right lung was cut into 6 (R–I to R–VI); B) the left lung was cut into 7 (L–I to L–VII) coronal slices, from ventral to dorsal. Twenty-two right sections (R1–R22) in R–IV and R–V and 20 left sections (L1–L20) in L–V and L–IV are shown in black boxes. The dotted white line is the boundary between the upper and lower lobes. The SARS-CoV-2 RNA score is indicated by the number of red circles and the *S. pneumoniae* DNA score is indicated by the number of yellow circles. (-) indicates results under the detection limit. Scale bars indicate 2 cm. SARS-CoV-2, severe acute respiratory syndrome coronavirus 2.

**Figure 2:**
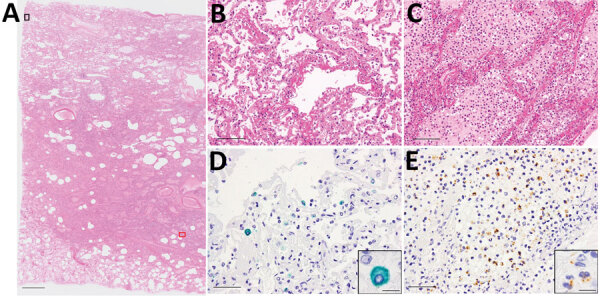
Microscopic findings of the lungs of a patient in Japan co-infected with SARS-CoV-2 and *Streptococcus pneumoniae*. A) Histopathology of lung section R12 (shown in Figure 1). Scale bar indicates 2 mm. B) Magnified image of the black square (top left) in panel A: exudative phase of diffuse alveolar damage (DAD) with hyaline membranes. Scale bar indicates 100 μm. C) Magnified image of the red square (bottom right) in panel A: edema and bronchopneumonia with massive infiltration of neutrophils in the alveolar spaces. Scale bar indicates 100 μm. D, E) Magnified images of the same areas of consecutive sections as B and C, respectively, showing SARS-CoV-2 antigen stained green (Vina green) and *S. pneumoniae* antigen stained brown (3,3′-diaminobenzidine) by enzyme-labeled double immunohistochemistry. The SARS-CoV-2 antigens were detected predominantly in the DAD area (D; scale bar indicates 50 μm). The *S. pneumoniae* antigens were detected predominantly in the bronchopneumonia area (E; scale bar indicates 50 μm). Insets show magnified images of the staining cells (scale bars indicate 10 μm).

The autopsy was conducted 45 hours after the patient’s death. Macroscopically, the lungs (left, 680 g; right, 800 g) were mostly colored red and consolidated with only remnant airspaces accompanied by a small pleural effusion. The heart (450 g) exhibited no macroscopic intravascular thrombosis. There were no remarkable changes in other organs, including the liver (1120 g), kidneys (left, 140 g; right, 100 g), and spleen (110 g). Microscopically, the epithelial cells of the trachea, bronchi, and bronchioles were mostly denuded, with submucosal inflammatory cell infiltration, edema, and congestion (Appendix Figure 2, panel A). Histological analysis of 42 lung sections (Figure 1) showed the acute exudative phase and early organizing phase of diffuse alveolar damage (DAD) with hyaline membrane formation (Figure 2, panels A, B; Appendix Figure 2, panel B). We observed edema with fibrin deposits, desquamated alveolar epithelial cells, mononuclear cell infiltrates, and multinucleated syncytial cells in the alveolar air spaces (Appendix Figure 2, panel C), and various degrees of inflammatory cell infiltration and edema in the interstitium. In addition, we observed neutrophil infiltration in the alveolar spaces scattered throughout the lower lobes, suggestive of acute bronchopneumonia (Figure 2, panels A, C; Appendix Figure 2, panel D). We noted a limited number of gram-positive cocci in the intracellular and extracellular regions (Appendix Figure 2, panel E). Vascular congestion was present in several lung sections with prominent fibrin microthrombi in blood vessels of various sizes (Appendix Figure 2, panel F). We did not see either endotheliitis or vasculitis with fibrinoid necrosis.

We determined the copy numbers of SARS-CoV-2 RNA and human glyceraldehyde 3-phosphate dehydrogenase mRNA in formalin-fixed paraffin-embedded tissue specimens by rRT-PCR, as previously described (*3*). We detected moderate or higher copy numbers of SARS-CoV-2 RNA in all lung sections. The ratios of SARS-CoV-2 RNA to glyceraldehyde 3-phosphate dehydrogenase mRNA in the upper lobes were significantly greater than those in the lower lobes (Mann-Whitney test: right lung, p<0.05; left lung, p<0.0001) (Table; Appendix Figure 3, panel A). We screened the microbial DNA in the formalin-fixed paraffin-embedded lung specimens using a multimicrobial rRT-PCR system that simultaneously detects 68 bacterial species and 9 fungal species (*4*). This screening yielded a positive result for *S. pneumoniae*, which was confirmed by rRT-PCR (*5*). The ratio of *S. pneumoniae* DNA to β-actin DNA (*6*) in the lower lobes was significantly greater than that in the upper lobes (Mann-Whitney test: right lung, p<0.005; left lung, p<0.0001) (Table; Appendix Figure 3, panel B). *S. pneumoniae* DNA was not detected in several lung sections in the upper lobes and the extrapulmonary tissues except for the pharynx and trachea, suggesting absence of bacteremia.

**Table:** Quantification of SARS-CoV-2 RNA and *Streptococcus pneumoniae* DNA in 42 lung sections from a patient in Japan co-infected with both pathogens*

Lung lobe	Lung section	SARS-CoV-2 RNA, copies/μL	GAPDH mRNA, copies/μL	SARS-CoV-2 RNA/GAPDH mRNA ratio	SARS-CoV-2 RNA score†	*S. pneumoniae* DNA, copies/μL	ACTB DNA, copies/μL	*S. pneumoniae* DNA/ACTB DNA ratio × 10^5^	*S. pneumoniae* DNA score‡
RUL	R1§	2.01 × 10^6^	1.31 × 10^3^	1,534	3	1.67 × 10^2^	5.66 × 10^4^	295	2
	R2	1.71 × 10^6^	1.03 × 10^3^	1,660	3	2.01 × 10^2^	8.24 × 10^4^	244	2
	R3	6.66 × 10^5^	1.66 × 10^3^	401	2	4.17 × 10^1^	3.40 × 10^4^	123	2
	R4	6.93 × 10^5^	1.70 × 10^3^	408	2	5.36 × 10^1^	4.58 × 10^4^	117	2
	R5	6.87 × 10^5^	1.80 × 10^3^	382	2	2.48 × 10^1^	7.39 × 10^4^	34	1
	R6	1.98 × 10^5^	1.21 × 10^3^	164	2	9.07 × 10^1^	1.33 × 10^5^	68	1
	R7	8.98 × 10^5^	1.65 × 10^3^	544	2	5.24 × 10^1^	1.02 × 10^5^	51	1
	R8	5.48 × 10^5^	8.66 × 10^2^	633	2	UDL	1.07 × 10^5^	UDL	0
RLL	R9	7.50 × 10^5^	8.82 × 10^2^	850	2	2.10 × 10^2^	7.33 × 10^4^	286	2
	R10	1.71 × 10^6^	1.31 × 10^3^	1,305	3	4.50 × 10^2^	1.02 × 10^5^	441	2
	R11	1.15 × 10^6^	1.08 × 10^3^	1,065	3	8.94 × 10^2^	6.72 × 10^4^	1,330	3
	R12	1.39 × 10^5^	8.85 × 10^2^	157	2	4.70 × 10^3^	6.33 × 10^4^	7,425	3
	R13	3.45 × 10^5^	9.42 × 10^2^	366	2	9.44 × 10^1^	8.59 × 10^4^	110	2
	R14	6.62 × 10^3^	5.55 × 10^2^	12	1	5.37 × 10^3^	6.98 × 10^4^	7,693	3
	R15	1.85 × 10^4^	9.14 × 10^2^	20	1	1.74 × 10^3^	9.09 × 10^4^	1,914	3
	R16	1.40 × 10^4^	8.20 × 10^2^	17	1	4.14 × 10^3^	1.03 × 10^5^	4,019	3
	R17	2.92 × 10^4^	8.58 × 10^2^	34	1	2.67 × 10^3^	6.54 × 10^4^	4,083	3
	R18	1.93 × 10^4^	8.04 × 10^2^	24	1	3.18 × 10^3^	8.22 × 10^4^	3,869	3
	R19	3.05 × 10^4^	6.46 × 10^2^	47	1	2.56 × 10^3^	6.00 × 10^4^	4,267	3
	R20	5.16 × 10^4^	6.81 × 10^2^	76	1	2.92 × 10^3^	5.92 × 10^4^	4,932	3
	R21	1.50 × 10^4^	5.82 × 10^2^	26	1	3.18 × 10^3^	8.53 × 10^4^	3,728	3
	R22	5.27 × 10^5^	5.88 × 10^2^	896	2	2.12 × 10^1^	4.57 × 10^4^	46	1
LUL	L1	5.44 × 10^6^	1.52 × 10^3^	3,579	3	UDL	7.39 × 10^4^	UDL	0
	L2	1.34 × 10^6^	1.91 × 10^3^	702	2	6.85 × 10^1^	1.20 × 10^5^	57	1
	L3	6.73 × 10^5^	1.78 × 10^3^	378	2	UDL	6.16 × 10^4^	UDL	0
	L4	1.52 × 10^5^	1.36 × 10^3^	112	2	2.00 × 10^1^	8.92 × 10^4^	22	1
	L5	9.59 × 10^5^	1.83 × 10^3^	524	2	UDL	4.99 × 10^4^	UDL	0
	L6	1.59 × 10^5^	1.33 × 10^3^	120	2	3.13 × 10^2^	4.26 × 10^4^	735	2
	L7	3.85 × 10^6^	1.94 × 10^3^	1,985	3	1.50 × 10^1^	6.69 × 10^4^	22	1
	L8	1.77 × 10^6^	2.36 × 10^3^	750	2	UDL	5.40 × 10^4^	UDL	0
	L9	1.02 × 10^6^	1.37 × 10^3^	745	2	UDL	4.96 × 10^4^	UDL	0
	L10	3.11 × 10^6^	2.22 × 10^3^	1,401	3	UDL	6.25 × 10^4^	UDL	0
LLL	L11	1.27 × 10^5^	1.07 × 10^3^	119	2	5.54 × 10^2^	4.49 × 10^4^	1,234	3
	L12	3.80 × 10^4^	8.48 × 10^2^	45	1	6.42 × 10^3^	1.12 × 10^5^	5,732	3
	L13	2.16 × 10^4^	7.91 × 10^2^	27	1	5.91 × 10^3^	8.87 × 10^4^	6,663	3
	L14	4.87 × 10^4^	8.73 × 10^2^	56	1	2.85 × 10^3^	7.76 × 10^4^	3,673	3
	L15	4.65 × 10^4^	1.22 × 10^3^	38	1	4.08 × 10^3^	6.36 × 10^4^	6,415	3
	L16	2.86 × 10^4^	8.88 × 10^2^	32	1	5.68 × 10^3^	6.80 × 10^4^	8,353	3
	L17	3.42 × 10^4^	9.42 × 10^2^	36	1	3.58 × 10^3^	6.45 × 10^4^	5,550	3
	L18	3.89 × 10^4^	9.47 × 10^2^	41	1	4.64 × 10^3^	6.87 × 10^4^	6,754	3
	L19	4.25 × 10^3^	5.81 × 10^2^	7	1	5.82 × 10^2^	1.02 × 10^5^	571	2
	L20	1.56 × 10^4^	8.09 × 10^2^	19	1	6.10 × 10^1^	9.11 × 10^4^	67	1

*ACTB, β-actin; GAPDH, glyceraldehyde 3-phosphate dehydrogenase; LLL, left lower lobe; LUL, left upper lobe; RLL, right lower lobe; RUL, right upper lobe; SARS-CoV-2, severe acute respiratory syndrome coronavirus 2; UDL, under detection limit. †SARS-CoV-2-RNA score is as follows: score 1, SARS-CoV-2 RNA/GAPDH mRNA ratio<100; score 2, 100–1,000; score 3, 1,000–10,000. ‡*S. pneumoniae* DNA score is as follows: score 1, *S. pneumoniae* DNA/ ACTB DNA ratio × 10^5^ <100; score 2, 100–1,000; score 3, 1,000–10,000.  §Lung sections R1–R22 and L1–L20) are shown in Figure 1.

We performed immunohistochemistry (IHC) using a rabbit polyclonal antibody against SARS-CoV-2 antigens (*7*). We detected a large number of viral antigen-positive cells in lung sections with high SARS-CoV-2 RNA scores (Figure 2, panel D; Appendix Figure 2, panels G, H). The distribution of SARS-CoV-2 spike RNA detected by in situ hybridization (*8*) was similar to that of the viral antigen (Appendix Figure 2, panel I). Double fluorescence staining for in situ hybridization and IHC detected both the viral RNA and viral antigen in the same cells (Appendix Figure 4, panels A–C). Double immunofluorescence staining revealed that SARS-CoV-2 antigens were present in epithelial membrane antigen–positive bronchiolar and alveolar epithelial cells and CD68-positive macrophages/monocytes (Appendix Figure 4, panels D–I). We found multiple fibrin microthrombi in several lung vessels, but we detected no viral antigen in CD34-positive vascular endothelial cells (data not shown). IHC using an antibody against *S. pneumoniae* spp*.* (NB100–64502; Novus Biologicals, https://www.novusbio.com) showed both intact streptococci and granular antigens staining in neutrophils, macrophages, or both, particularly in the lesion with bronchopneumonia (Figure 2, panel E).

Of note, the copy numbers of viral RNA and bacterial DNA in each lung section were found to be inversely correlated, suggesting that the viral and bacterial infections occurred in a mutually exclusive manner in the lung tissues (Figure 1; Appendix Figure 5). Enzyme-labeled double IHC detected only viral antigens in areas of DAD and only bacterial antigens in bronchopneumonia lesions, similar to the findings in the whole lungs (Figure 2, panels D, E). Although it is unknown whether SARS-CoV-2 infection preceded, coincided with, or followed *S. pneumoniae* infection, it can be assumed that the patient developed acute respiratory distress syndrome induced by COVID-19 pneumonia and had concomitant bronchopneumonia caused by *S. pneumoniae* infection.

We found no notable changes in the extrapulmonary tissues related to COVID-19, including thrombosis. Although low copy numbers of SARS-CoV-2 RNA were detected in the pharynx, trachea, and intestines, we detected no viral antigens.

## Conclusions

The patient, who died on the eighth day of illness, had mostly acute-phase DAD with overwhelming viral infection, as demonstrated by detection of high titers of viral RNA and antigens in the lung sections. These results indicate a relatively early phase of SARS-CoV-2 infection, which implies that bacterial co-infection may have contributed to an abrupt deterioration of respiratory function in the patient. Bacterial co-infection of the respiratory tract has been well characterized in influenza, with a reported co-infection rate exceeding 30% in hospitalized patients (*9**,**10*). Co-infection with *S. pneumoniae* and *Staphylococcus aureus* has been linked to excess illness and death (*9*). In contrast, recent studies have suggested that bacterial co-infection is relatively uncommon in patients with COVID-19, with a prevalence of 3.5% in patients who were newly admitted to the hospital (*11**,**12*). However, given the serious and potentially lethal complications resulting from bacterial infections, the possibility of co-infection with other microbial pathogens should be also considered in patients with COVID-19, especially in elderly patients with severe disease, and it is difficult to identify bacterial co-infection on computed tomography images alone after the development of acute respiratory distress syndrome (*13*).

In conclusion, in-depth postmortem examination revealed that SARS-CoV-2 and *S. pneumoniae* had differential intrapulmonary distribution in this patient, independently causing DAD and bronchopneumonia pathology. Patients with COVID-19 should be evaluated carefully for co-infection with other pathogens to fully understand the effect of co-infection on COVID-19 pathology.

Appendix Additional information about lung pathology of mutually exclusive co-infection of SARS-CoV-2 and *Streptococcus pneumoniae*.
