# Supplementary material for: Lung Pathology of Mutually Exclusive Co-infection with SARS-CoV-2 and Streptococcus pneumoniae
Source: Emerg Infect Dis. 2021 Mar;27(3):919–23. doi: 10.3201/eid2703.204024 (PMC7920656; doi:10.3201/eid2703.204024)
Supplement: Appendix — Additional information about lung pathology of mutually exclusive co-infection of SARS-CoV-2 and Streptococcus pneumoniae. [file 20-4024-Techapp-s1.pdf]

Article DOI: https://doi.org/10.3201/eid2703.204024

Lung Pathology of Mutually Exclusive Co-infection with SARS-CoV-2 and *Streptococcus pneumoniae*

Appendix

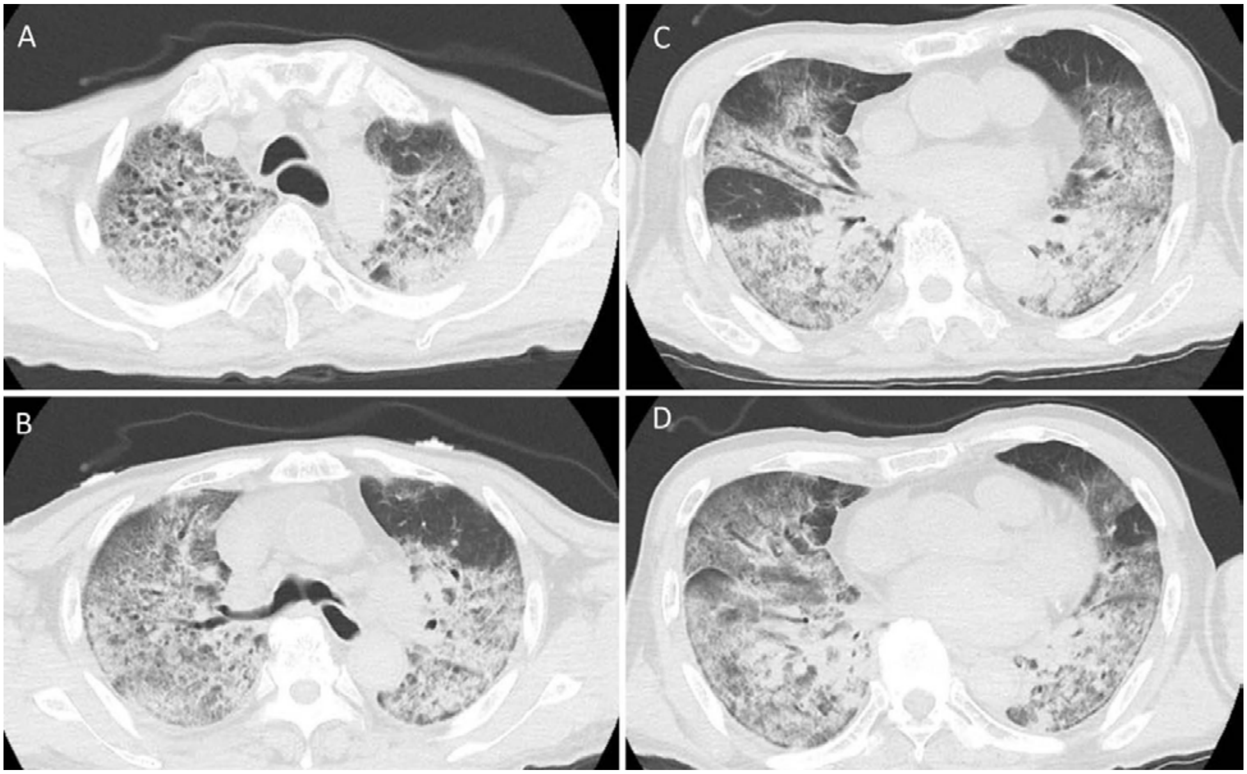

Appendix Figure 1. Computed tomography (CT) chest imaging performed in the emergency department: A) upper lobes, B) carina, C) middle lobe of right lung, and D) lower lobes. Axial CT image shows diffuse bilateral extensive consolidation with ground-glass opacities, with intralobular septal thickening prominent in the upper lobes. The relative subpleural sparing is evident. These findings were more compatible with acute respiratory distress syndrome (ARDS); it was difficult to differentiate the CT findings of viral infection from those of bacterial bronchopneumonia.

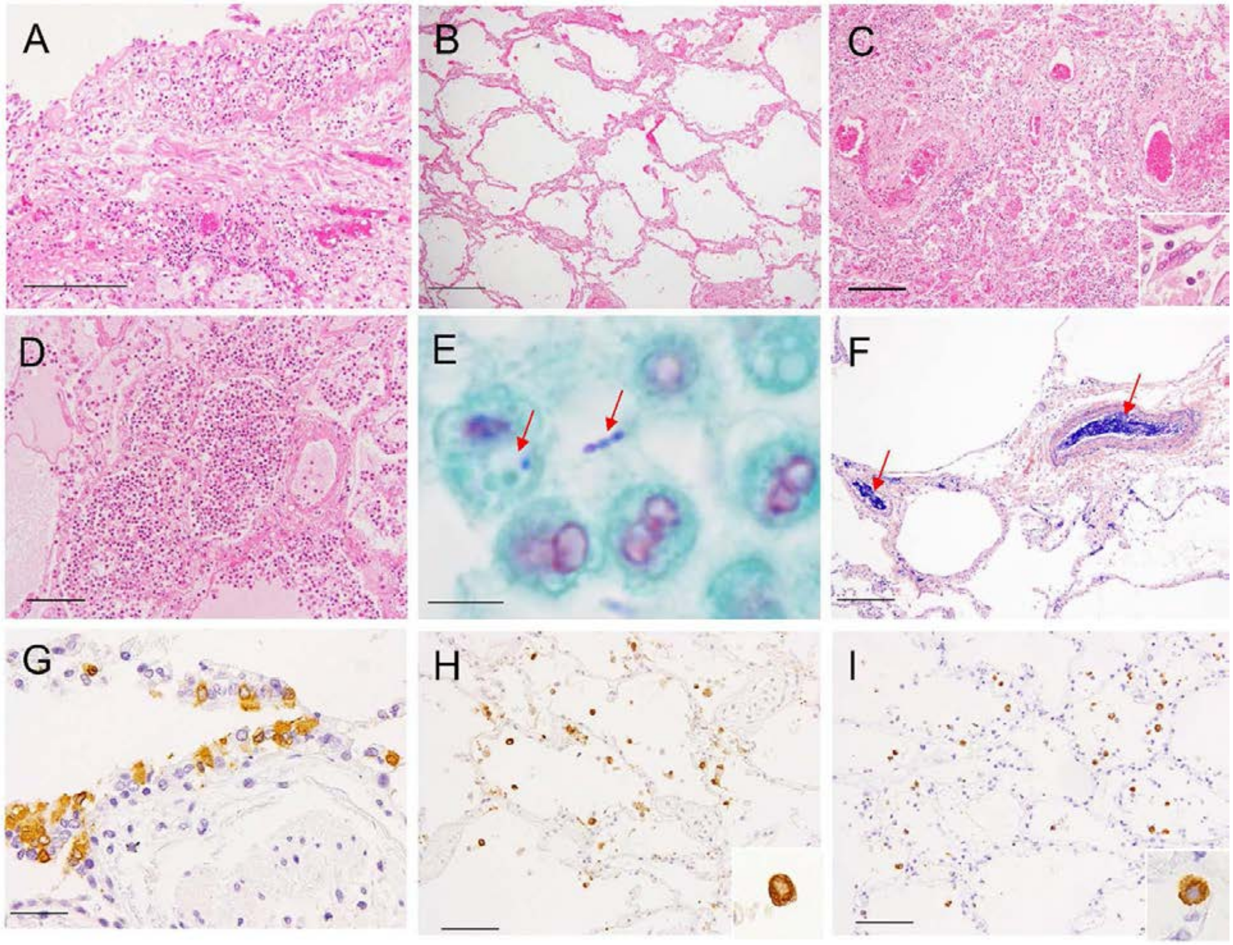

Appendix Figure 2. Histopathology, immunohistochemistry (IHC), and in situ hybridization (ISH) of the lungs: A) Bronchitis with submucosal inflammatory cell infiltration, edema, and congestion. Scale = 200 μm. B) Acute phase of diffuse alveolar damage (DAD) with hyaline membranes. Scale = 500 μm. C) Early organizing phase of DAD with interstitial inflammation and desquamation of the epithelium in the alveolar space and a multinucleated syncytial cell (inset). Scale = 200 μm. D) Edema and bronchopneumonia with massive infiltration of neutrophils in the alveolar spaces. Scale = 100 μm. E) Gram/Twort staining. Extracellular gram-positive cocci were observed in chains and a coccus in cytosol (red arrows). Scale = 5 μm. F) Fibrin microthrombi were confirmed in blood vessels of various sizes by phosphotungstic acid hematoxylin (PTAH) staining (purple, red arrows). Scale = 200 μm. G) IHC for viral antigen (brown) in the bronchial epithelium. Scale = 50 μm. H) IHC for viral antigen (brown) in the alveolar epithelium and macrophages. Scale = 100 μm. I) ISH of SARS-CoV-2 spike RNA (brown). Scale = 100 μm. Insets: magnified images of the staining cells.

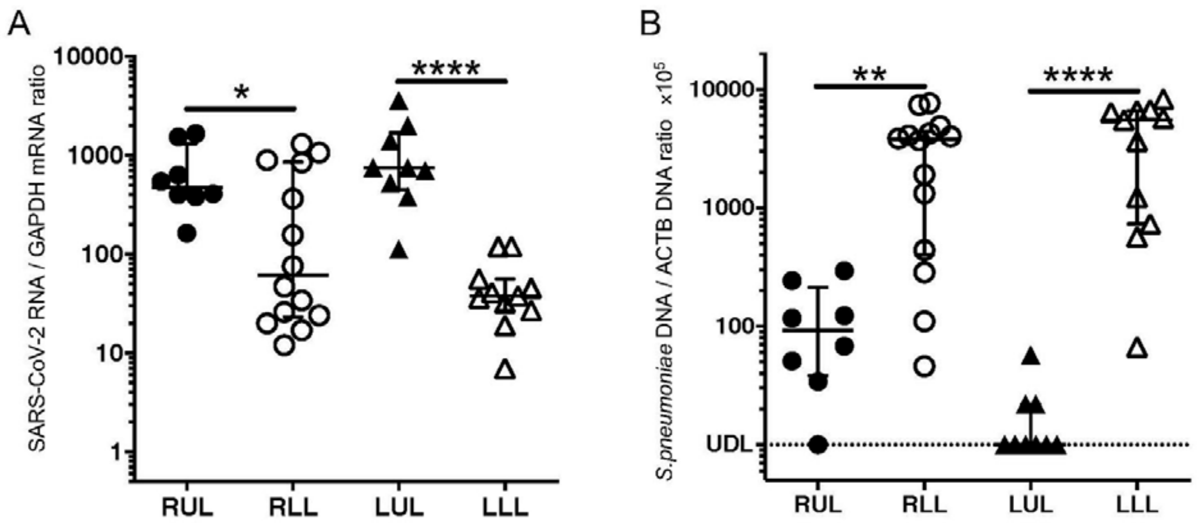

Appendix Figure 3. The amount of SARS-CoV-2 RNA and *S. pneumoniae* DNA detected in each lung lobe. A) Distribution of SARS-CoV-2 RNA/GAPDH mRNA ratio in the right upper lobe (RUL), right lower lobe (RLL), left upper lobe (LUL), and left lower lobe (LLL). Differences between upper and lower lobes were analyzed using the Mann-Whitney U test. Significant differences: *p<0.05, ****p<0.0001. B) Distribution of the pneumococcus DNA/ACTB DNA ratio $\times 10^5$ in the right upper lobe (RUL), right lower lobe (RLL), left upper lobe (LUL), and left lower lobe (LLL). Differences between upper and lower lobes were analyzed using the Mann-Whitney U test. Significant differences: **p<0.005, ****p<0.0001. ACTB, β-actin; GAPDH, glyceraldehyde 3-phosphate dehydrogenase.

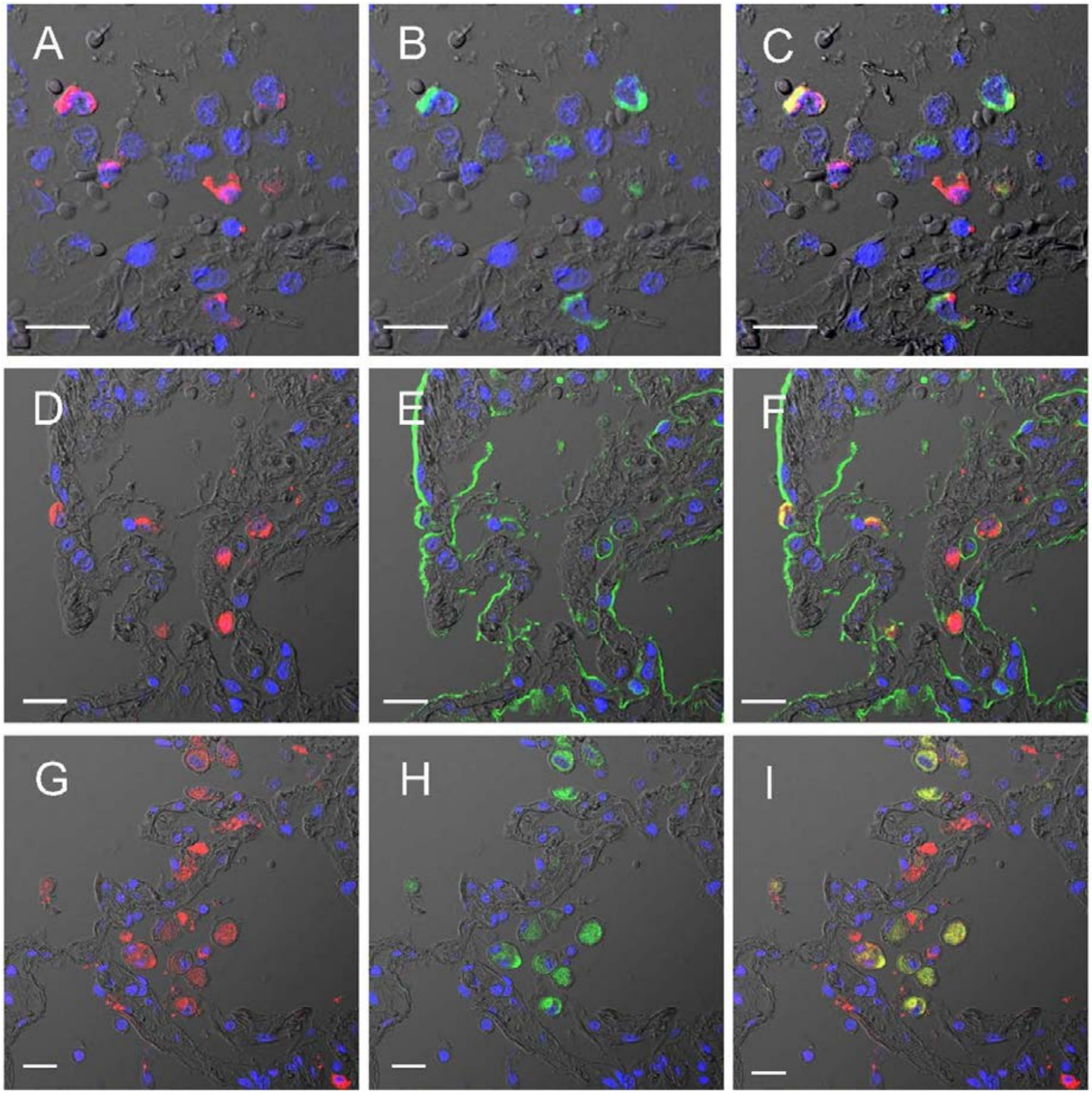

Appendix Figure 4. A–C) Double fluorescence staining for in situ hybridization (ISH) and immunohistochemistry (IHC), and D–I) double immunofluorescence staining. A) The signals for IHC for SARS-CoV-2 antigen (red). B) ISH for SARS-CoV-2 spike RNA (green). C) Merged images of A and B. D–I) Double immunofluorescence staining for SARS-CoV-2 antigen (D, G; red) with epithelial cell marker (E; EMA, green) and macrophage marker (H; CD68, green). F) Merged images of D and E. I) Merged images of G and H. TO-PRO-3 nucleic acid staining (blue) and differential interference contrast (DIC) images are also shown. Scale = 20 μm.

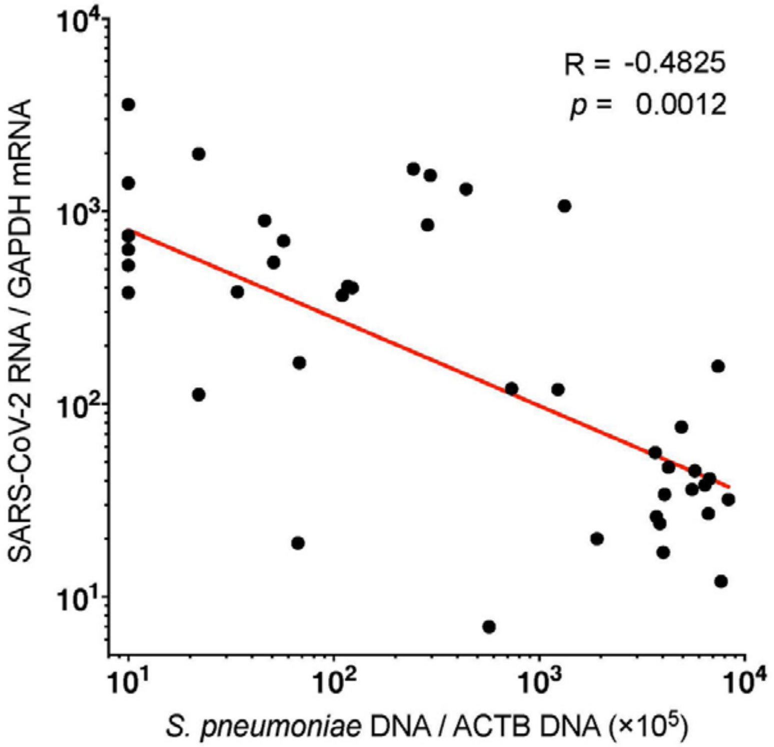

Appendix Figure 5. The SARS-CoV-2 RNA/GAPDH mRNA ratio and *S. pneumoniae* DNA/ACTB DNA ratio $\times 10^5$ were inversely correlated (correlation coefficient −0.4825, p = 0.0012). ACTB, β-actin; GAPDH, glyceraldehyde 3-phosphate dehydrogenase.
